# mRNA-seq and miRNA-seq profiling analyses reveal molecular mechanisms regulating induction of fruiting body in *Ophiocordyceps sinensis*

**DOI:** 10.1038/s41598-021-91718-x

**Published:** 2021-06-21

**Authors:** Han Zhang, Pan Yue, Xinxin Tong, Jing Bai, Jingyan Yang, Jinlin Guo

**Affiliations:** 1 Key Laboratory of Standardization of Chinese Medicine, Ministry of Education; Key Laboratory of Systematic Research of Distinctive Chinese Medicine Resources in Southwest China, Resources Breeding Base of Co-Founded By Sichuan Province and MOST, Chengdu University of Traditional Chinese Medicine, Chengdu, 611137 China; 2 State Key Laboratory of Grassland Agro-Ecosystem, Institute of Innovation Ecology, Lanzhou University, Lanzhou, 730000 China

**Keywords:** Gene expression, Gene regulation, Genetics, Molecular biology

## Abstract

*Ophiocordyceps sinensis* has been a source of valuable materials in traditional Asian medicine for over two thousand years. With recent global warming and overharvest, however, the availability of these wild fungi has decreased dramatically. While fruiting body of *O. sinensis* has been artificially cultivated, the molecular mechanisms that govern the induction of fruiting body at the transcriptional and post-transcriptional levels are unclear. In this study, we carried out both mRNA and small RNA sequencing to identify crucial genes and miRNA-like RNAs (milRNAs) involved in the development of fruiting body. A total of 2875 differentially expressed genes (DEGs), and 71 differentially expressed milRNAs (DEMs) were identified among the mycoparasite complex, the sclerotium (ST) and the fruiting body stage. Functional enrichment and Gene Set Enrichment Analysis indicated that the ST had increased oxidative stress and energy metabolism and that mitogen-activated protein kinase signaling might induce the formation of fruiting body. Integrated analysis of DEGs and DEMs revealed that n_os_milR16, n_os_milR21, n_os_milR34, and n_os_milR90 could be candidate milRNAs that regulate the induction of fruiting body. This study provides transcriptome-wide insight into the molecular basis of fruiting body formation in *O. Sinensis* and identifies potential candidate genes for improving induction rate.

## Introduction

*Ophiocordyceps sinensis* (Berk.) G. H. Sung, J. M. Sung, Hywel‐Jones & Spatafora consists of a sclerotium, holding the carcass of Hepialidae insect larvae, and stroma, the fruiting body of which is also the primary fungal structure used for taxonomic identification^1,2^. As one of the most valuable fungal traditional medicinal materials, *O. sinensis* has been widely used to treat lung inflammation, night sweats, asthma, nocturnal emissions, and other diseases for over two thousand years^3,4^. Due to environmental conditions, the fruiting body of *O. sinensis* forms only after 3–5 years in the wild^5^. In recent years, overharvest due to huge market demand has led to the rapid reduction of wild *O. sinensis* populations, promoting the need for and development of artificial cultivation of *O. sinensis*. Even under optimized artificial cultivation conditions, it takes the fruiting body more than one year to mature due to its complex life cycle^6–8^. However, the induction of fruiting body is still inefficient, and the high cost of large-scale artificial cultivation of this fungus is unsustainable in China.

The development of the fruiting body inside ascomycetes is a complex cellular differentiation process that requires special environmental conditions and is controlled by many developmentally-regulated genes. With the advancement of whole-genome data of *O. sinensis*, the molecular mechanisms of its growth and development have gradually been revealed^9,10^. Four mating-type genes and 121 other genes that may be involved in fruiting body development were discovered via the *O. sinensis* 454-EST database, which indicated that the mitogen-activated protein kinase (MAPK) signaling pathway was likely involved in the development of fruiting body^11^. Recently, transcriptome analyses have compared the transcripts of six *O. sinensis* developmental stages^12^, suggested that fungi in primordium differentiation and sexual maturation display similar gene expression patterns. Moreover, half of the genes related to mating showed the highest expression in the ST stage, indicating that fruiting in this fungus is initiated in the ST stage^12^. Previously, we compared transcript expression in three other stages of the fungal life cycle (asexual mycelium, developing fruiting body, and mature fruiting body). Four fifths MAPKKK genes and MAPK binding proteins were upregulated in the fruiting body compared with the mycelium, indicating the development of fruiting body in *O. sinensis* may be dependent on the MAPK signaling pathway^13^. These results provided initial indications for further study of the mechanism of induction.

MicroRNAs (miRNAs) are a class of endogenous small single-stranded RNAs that are composed of approximately 19–24 nucleotides, and which play important roles in post-transcriptional regulation of gene expression in eukaryotes^14^. Although there are still no miRNAs identified in miRbase that are encoded by fungi, novel miRNA-like RNAs (milRNAs) have been predicted in the *O. sinensis* genome^15,16^. Recently, some studies have shown that small RNAs play vital roles in fungal sexual development. The small RNA-mediated RNA interference mechanism plays an important role in the fine-tuning of the transcriptome during ascospore formation in *Fusarium graminearum*^17^. In *Cordyceps militaris*, disruption and overexpression of candidates milR4 and milR16 confirmed that milRNAs in *C. militaris* regulate fruiting body formation^18^. These studies suggested that milRNAs may play important roles in the regulation of development in *O. sinensis*.

**Figure 1:**
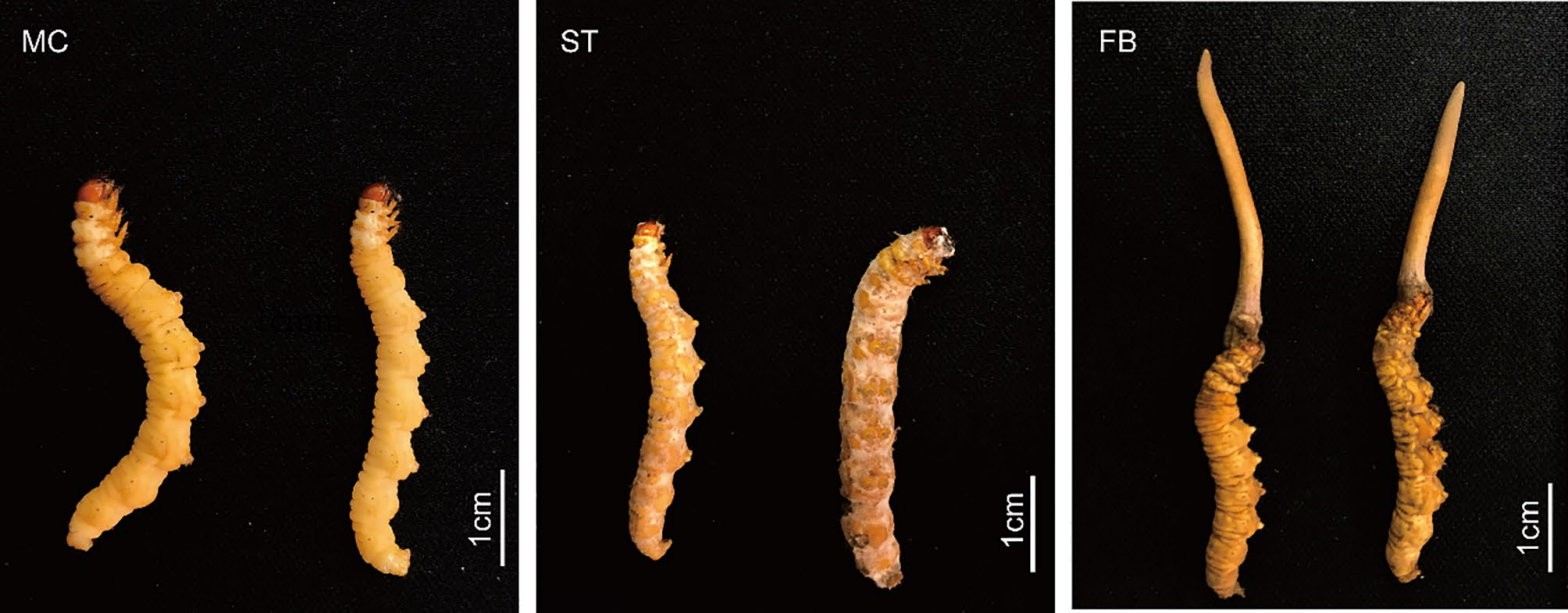
The morphologies of *O. sinensis* at three different developmental stages (*MC* mycoparasite complex, *ST* sclerotium, *FB* fruiting body).

In this study, three important stages of *O. sinensis* fruiting body formation were examined by RNA sequencing data. An integrated mRNA and miRNA transcriptome analysis was conducted before and after the sclerotium developmental stage (Fig. 1). Important factors and target genes associated with fruiting body induction during the development of *O. sinensis* were identified, providing a basic molecular mechanism to aid in facilitating large-scale artificial cultivation of *O. sinensis.*

## Results

### Overview of transcriptome and small RNA sequencing

**Figure 2:**
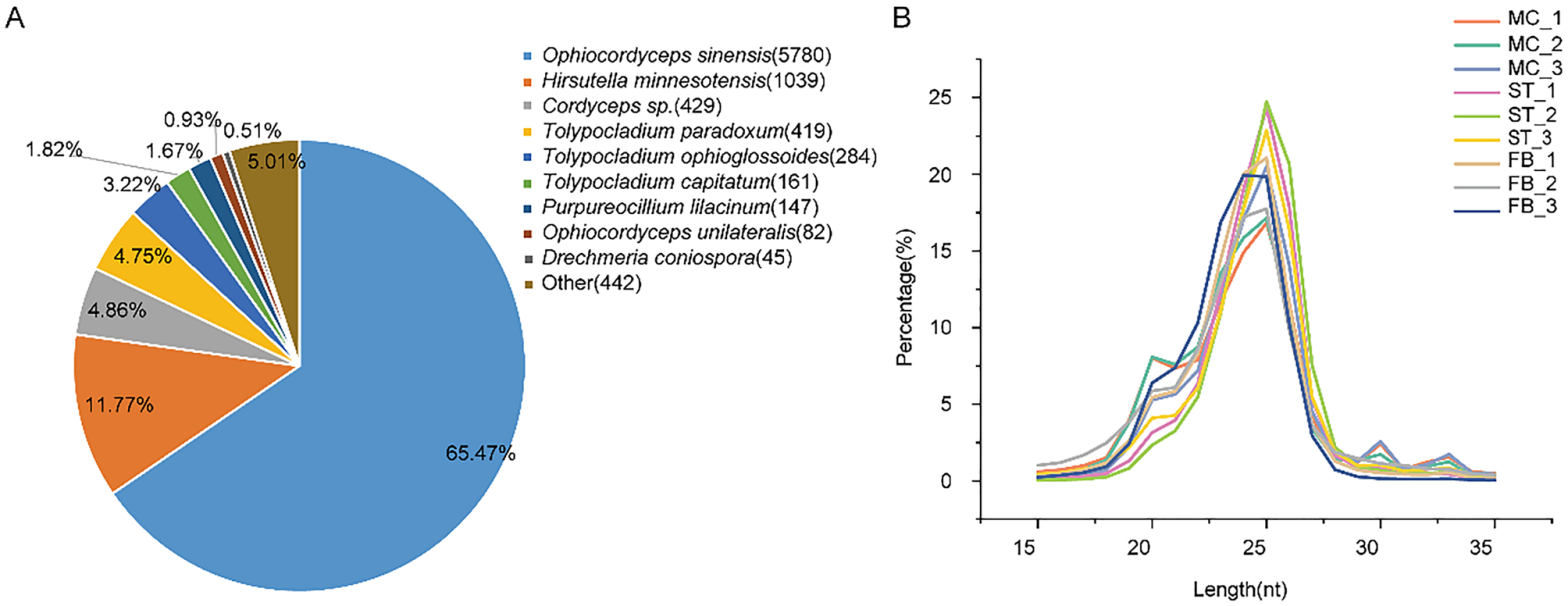
(**A**) Number of genes annotated in NR databases. (**B**) Length distribution and frequency of small RNAs (sRNAs) in the nine *O. sinensis* libraries.

After transcriptome sequencing and filtering, approximately 63.92 Gb of clean reads were obtained, with an average of 6.54 Gb for each sample from nine cDNA libraries, for which the Q30 base percentage was greater than 93.54% (Table S1). The clean reads from each sample were compared with the specified reference genome, for which the efficiency of alignment varied from 91.52 to 97.59%. The above results indicate that the sequences obtained in this study was of good quality and could be used for subsequent analysis. A total of 9921 transcripts were obtained, including 1008 that mapped to predicted new genes. Gene function annotations showed that 8851 genes had significant matches in COG, GO, KEGG, KOG, Pfam, Swissport, eggNOG, or NR databases, with 3241 (36.62%), 6129 (69.25%), 3359 (37.95%), 4490 (50.73%), 6361 (71.87%), 5298 (59.86%), 7962 (89.96%), and 8837 (99.84%) genes, respectively. In addition to the functional annotation of *O. sinensis*, 11.77% of the genes also had high homology with *Hirsutella minnesotensis* (Fig. 2A)*.*

After removing adaptors and low-quality reads, 142.96 M clean reads of small RNAs were generated, with each sample yielding greater than 11.05 M. The statistical results are shown in Table S2 with an overview of small RNA classification and annotation. The normalized clean reads were used for the analysis of small RNA distribution; the length distribution map of small RNA sequences demonstrated that the length of these small RNAs was 15–35 nucleotides (nt) (Fig. 2B). In general, most of the clean reads were 23–26 nt in length, with reads of 25 nt being the highest.

### DEGs and DEMs expression analysis of *O. sinensis* at differential development stages

**Figure 3:**
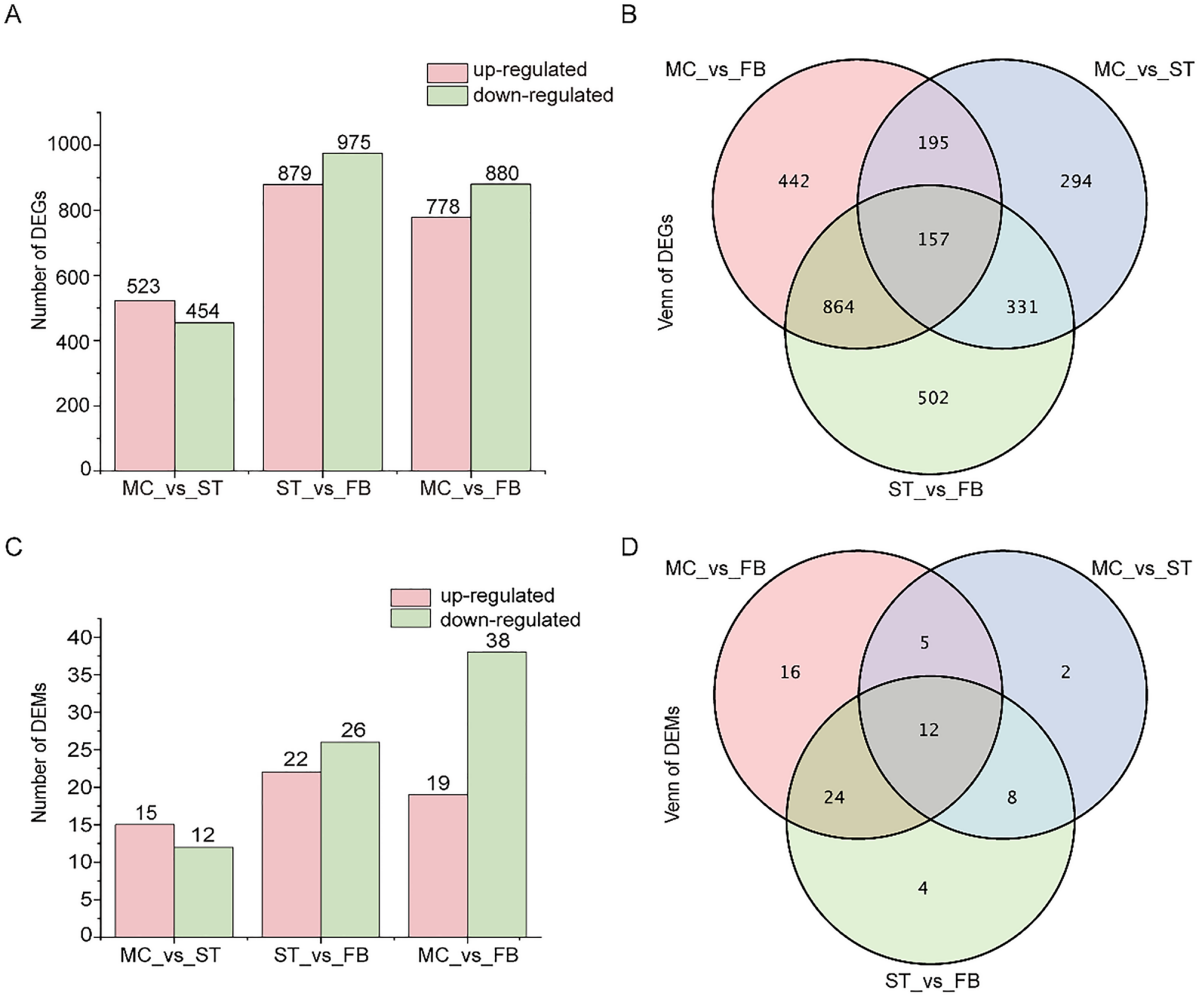
Comparative analysis of differentially expressed genes (DEGs) and milRNA (DEMs) at the MC, ST, and FB stage. (**A**) Numbers and (**B**) Venn diagram of DEGs between two samples (MC_vs_ST, ST_vs_FB, ST_vs_FB). (**C**) Numbers and (**D**) Venn diagram of DEMs between two samples (MC_vs_ST, ST_vs_FB, ST_vs_FB).

To investigate the changes in gene expression levels in *O. sinensis* during the development of fruiting body, DESeq2 software was used to compare the gene expression of samples at different stages based on clean reads. In the three comparison groups, we identified a total of 2875 DEGs. The initial stage (mycoparasite complex, MC) represented a control condition, the numbers of DEGs in the sclerotium (ST) stage and fruiting body (FB) periods were 977 and 1658, respectively. 1854 DEGs were also screened between the ST and FB stages (Fig. 3A). There were only 157 co-expressed genes in all three stages, and the most significant gene changes occurred during the FB stage (Fig. 3B). These differential genes included Cytochrome P450 monooxygenase (gene-G6O67_005633), Catalase (gene-G6O67_006909), Glucokinase (gene-G6O67_001528), and Phosphoenolpyruvate carboxykinase (gene-G6O67_008067) (Table S3), which are key enzyme genes in many metabolic pathways.

To investigate the known and putatively novel miRNAs expressed at the three stages of *O. sinensis*, we first compared the known mature miRNAs and miRNA precursors in miRBase; no conserved miRNAs were identified. However, a total of 106 novel milRNAs were identified in the nine small RNA libraries using the miRDeep2 program (Table S4). Differential expression analysis of the miRNAs between these three samples was performed based on normalized read counts (TPM) for each identified miRNA. We obtained 27, 48, and 57 differentially expressed milRNAs (DEMs) in MC vs ST, ST vs FB, and MC vs FB comparisons, respectively (Fig. 3C). More DEMs were downregulated during fruiting body development. Only 12 DEMs were co-expressed in all three stages. Characterizing the differential expression of miRNAs is important in predicting the occurrence and development of fruiting body in *O. sinensis* (Fig. 3D).

### Functional annotation and classification of DEGs

**Figure 4:**
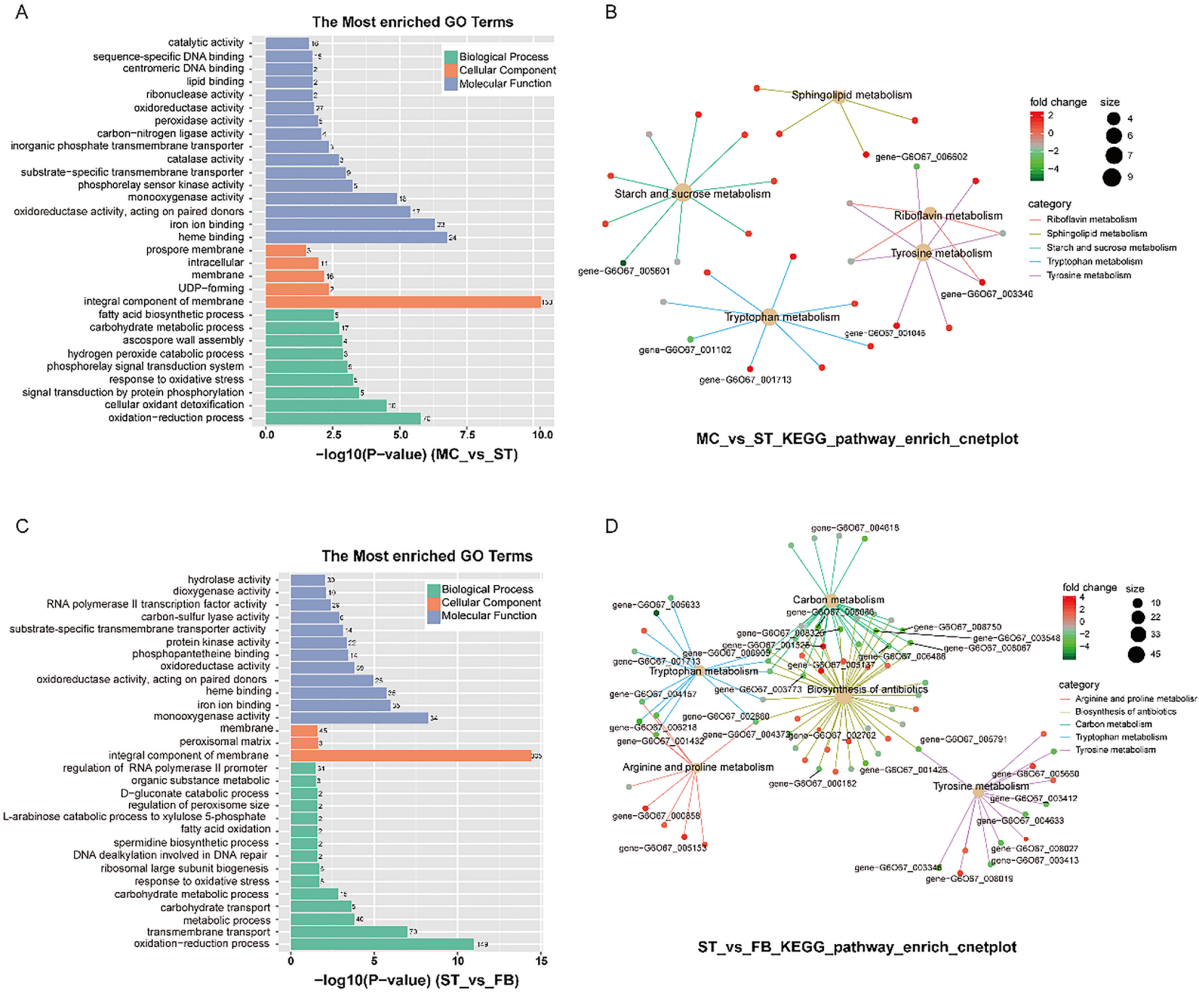
Gene Ontology and KEGG pathway enrichment of DEGs. (**A**) The most enriched GO terms and (**B**) KEGG pathway cnetplot of MC_vs_ST. (**C**) The most enriched GO terms and (**D**) KEGG pathway cnet plot of ST_vs_FB (GO *P *value ≤ 0.03, top five KEGG pathway category, the shown genes log2|FC|≥ 2).

To infer the biological functions affected by DEGs at the three stages (MC, ST, and FB), we performed GO functional analysis. In the two developmental processes, 477 and 1027 DEGs were classified into 47 terms of three major biological processes (biological processes, cellular components, and molecular functions), respectively. The enrichment results of the three major biological functions of GO are shown in Fig. 4A,B (*P *value ≤ 0.03). The most dominant terms included the oxidation–reduction process, integral components of membranes, oxidoreductase activity, monooxygenase activity, and iron ion binding. KEGG pathway enrichment analysis was also conducted; 97 and 220 DEGs were enriched, corresponding to 77 and 103 pathways, respectively. In the process of ossification of *O. sinensis*, “Starch and sucrose metabolism”, “Tryptophan metabolism”, “Tyrosine metabolism”, and “Sphingolipid metabolism” pathways were significantly enriched (Fig. 4C). In the FB formation stage, the degree of enrichment of “Biosynthesis of antibiotics” and “Carbon metabolism” varied greatly (Fig. 4D). These metabolic pathways may closely relate to the formation of sclerotia and fruiting body. All DEGs, as well as GO and KEGG analysis results, are shown in Table S3. However, some DEGs encoded functionally unknown proteins, which might relate to *O. sinensis* growth and development; further studies will be required to verify their functionalities.

### Analysis of GSEA significant enrichment

**Figure 5:**
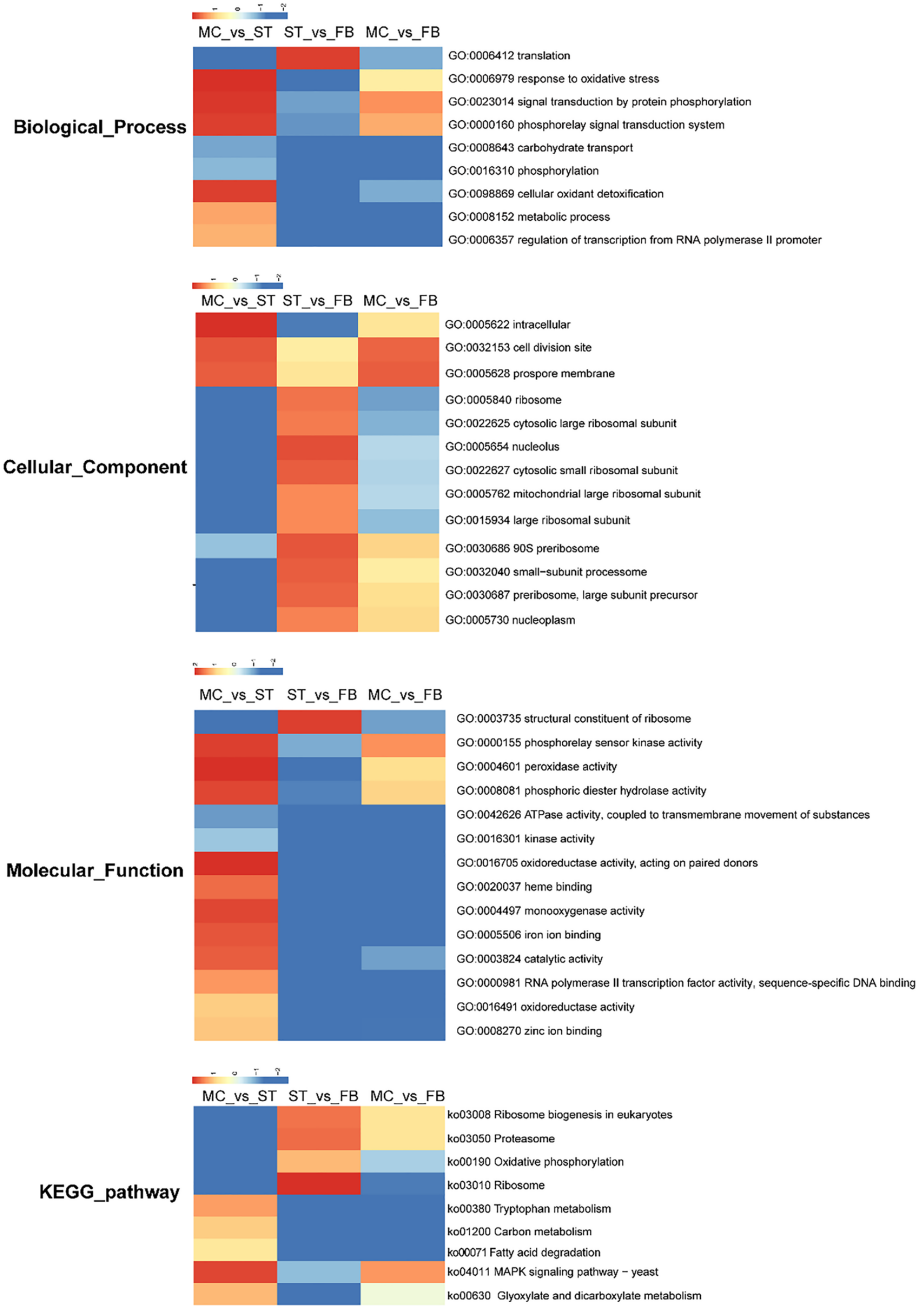
GSEA analysis heat map of *O. sinensis* at different development stages, include the biological process, cellular component, molecular function, and KEGG pathway (*P* < 0.05).

While GO- and KEGG-based analyses have compared the DEGs from functional categories, effective statistical methods were not used to analyze the overall trend of gene expression. Therefore, GSEA was adapted to analyze the enrichment of genes differentially expressed in each GO term and KEGG pathway. In this study, normalized enrichment scores were used to draw a cluster heat map (*P *value < 0.05) (Fig. 5). In the process of sclerotium formation, phosphorylation-related signaling (phosphorelay signal transduction system (GO:0000160), signal transduction by protein phosphorylation (GO:0023014), phosphorelay sensor kinase activity (GO:0023014), and oxidative phosphorylation (ko00190)) and oxidation-related (response to oxidative stress (GO:0000155), cellular oxidant detoxification (GO:0098869) peroxidase activity (GO:0004601), and monooxygenase activity (GO:0004497)) were significantly upregulated. During the fruiting body growth stage, the expression trend of ribosome-related terms and pathways were increased significantly, including the nucleolus (GO:0005730), ribosome (GO:0005840, ko030100), 90S preribosome (GO:0030686), and ribosome biogenesis in eukaryotes (ko03008). However, carbohydrate transport (GO:0008643), fatty acid degradation (ko00071), glyoxylate and dicarboxylate metabolism (ko00630), and carbon metabolism related to energy metabolism were downregulated.

Some prominent pathways are listed in Table 1, such as the MAPK signaling pathway. DEGs in this pathway include mitogen-activated protein kinase kinase (*pbs2*), glycerol-3-phosphate dehydrogenase (*gld1*), catalase (*cat1*), and other important genes encoding enzymes. The expression of Cat was upregulated with log_2_(fold change) of 2.312 and downregulated with log_2_(fold change) of 2.160, respectively. In the citrate cycle, the genes encoding the enzymes malate dehydrogenase (*mdh1*), phosphoenolpyruvate carboxykinase (*pck1*), and succinate-CoA ligase (*lsc2*), which catalyze the oxidation of citric acid for energy, were highest in the ST stage, upregulated with log_2_(FC) of 2.237, 3.607, and 3.025, respectively, compared with the FB stage, and were slightly higher than in the MC stage.

**Table 1:** DEGs involved in metabolic pathways. Significant differences in expression were evaluated using one-way ANOVA (ST was used as the control group, *P < 0.01).

Gene ID	Annotation	FPKM (Average value)		
		MC	ST	FB
**MAPK-related genes**				
gene-G6O67_001149	ATF/CREB family transcription factor	159.450	296.989	310.307
gene-G6O67_001860	Mitogen-activated protein kinase kinase	38.493	72.839	117.395
gene-G6O67_006243	Histidine kinase	35.787	77.025	31.850
gene-G6O67_006845	Pheromone receptor	10.897	33.218	15.188
gene-G6O67_005494	Glycerol-3-phosphate dehydrogenase (NAD +)	105.461	88.457	16.160*
gene-G6O67_007807	Ras homolog gene family	7.340	12.113	5.378*
gene-G6O67_001713	Catalase	290.274*	1624.276	362.997*
**Amino acid-related genes**				
gene-G6O67_003346	Tyrosinase	29.075*	427.065	1.401*
gene-G6O67_004157	Acetamidase	36.780*	94.229	23.372*
gene-G6O67_001432	Amidase signature domain protein	74.558	36.214	4.771*
gene-G6O67_006218	Amidase	84.452	101.630	4.214*
gene-G6O67_000733	Acetyl-CoA acetyltransferase	153.290	166.490	50.872*
gene-G6O67_001178	Glutaryl-CoA dehydrogenase	57.967	66.856	33.046
gene-G6O67_002860	Aldehyde dehydrogenase family	645.414*	1378.017	351.529*
gene-G6O67_005633	Cytochrome P450 monooxygenase	88.415	163.465	2.668*
gene-G6O67_001713	Catalase	290.274*	1624.276	362.997*
gene-G6O67_006145	Catalase-peroxidase	251.388	239.481	92.286*
**Energy-related metabolism genes**				
gene-G6O67_000383	Isocitrate dehydrogenase	87.656	48.958	102.003*
gene-G6O67_003773	Isocitrate lyase	42.254*	67.603	13.513*
gene-G6O67_001528	Glucokinase	17.177	11.438	176.097*
gene-G6O67_001884	Transaldolase	259.266	281.600	113.116*
gene-G6O67_003014	Acyl-CoA dehydrogenase	73.135	72.973	37.604
gene-G6O67_003548	Citrate synthase	326.182	61.291	0.000
gene-G6O67_003742	NAD(P)-binding domain protein	140.674	148.164	67.100
gene-G6O67_006486	Malate dehydrogenase	788.497	1088.906	236.372*
gene-G6O67_006510	Isocitrate dehydrogenase NADP	63.750	30.549	72.962
gene-G6O67_007408	Succinate dehydrogenase cytochrome b small subunit	269.799	242.459	111.577
gene-G6O67_007964	Acyl-CoA dehydrogenase domain protein	77.659	45.968	17.089
gene-G6O67_008750	Succinyl-CoA synthetase beta chain	87.758*	209.074	26.275*
gene-G6O67_008752	Alpha subunit of GDP-forming succinate-CoA ligase	118.068	145.249	42.014*
gene-G6O67_002224	Succinate dehydrogenase iron-sulfur protein	281.527	115.061	248.025
gene-G6O67_004996	Gluconolactonase precursor	16.812	5.929	8.262*
gene-G6O67_008325	Fructose-1,6-bisphosphatase I	140.215	107.728	8.231
gene-G6O67_008067	Phosphoenolpyruvate carboxykinase	929.438	1282.465	96.630*

### Integrated analysis of DEGs and DEMs

**Figure 6:**
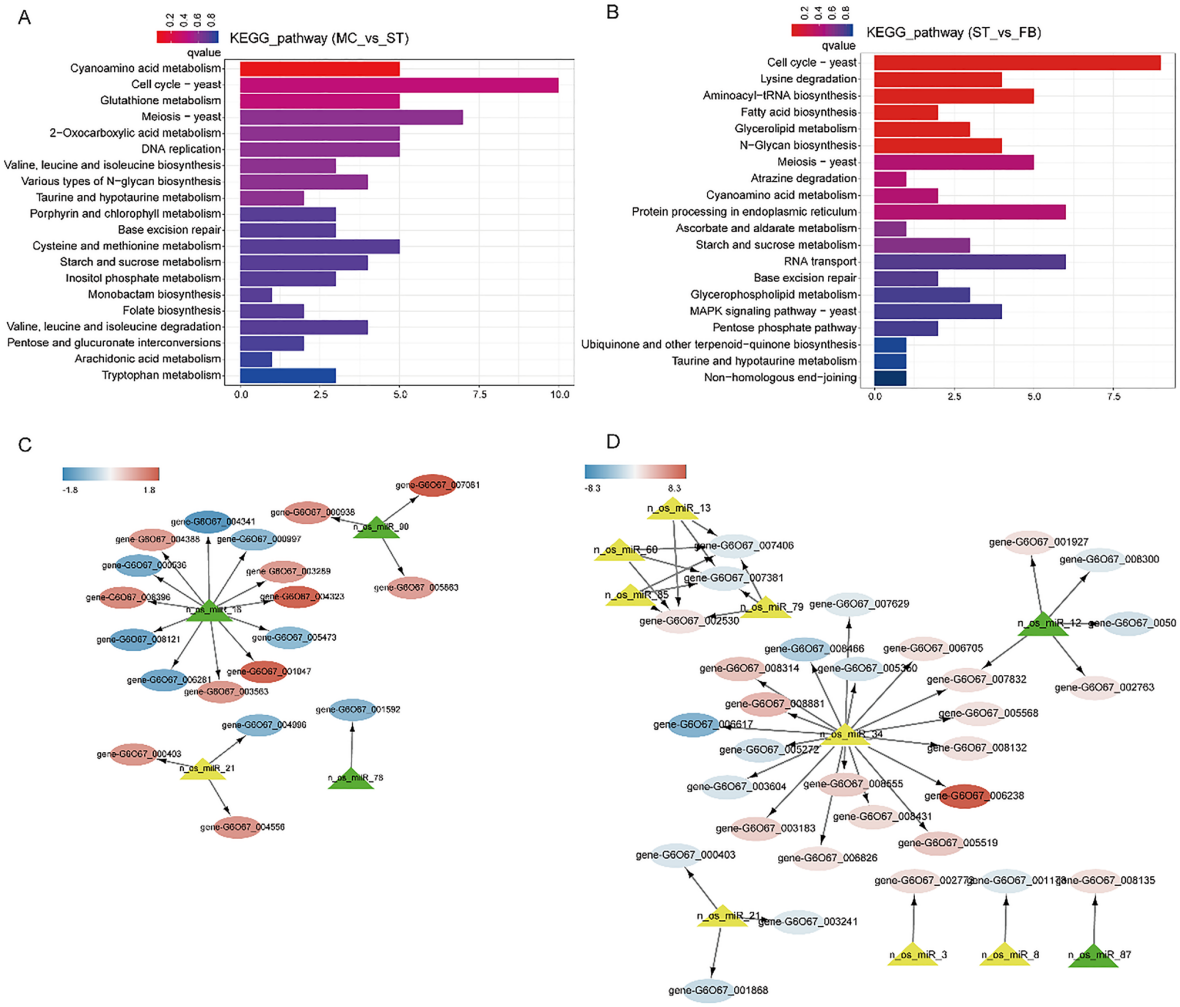
(**A**, **B**) KEGG enrichment analysis of differentially expressed genes (DEGs) targeted by differentially expressed milRNAs (DEMs). The regulatory relationship between DEMs and their targets DEGs in *O. sinensis* was determined. (**C**, **D**) The interaction network between DEMs and their differentially expressed targets (MC_vs_ST and ST_vs_FB). Circles indicate target genes, yellow triangles indicate upregulated milRNAs, and green triangles indicate downregulated milRNAs.

To explore the regulatory relationship between milRNAs and mRNAs, 1096 potential target genes of the milRNAs were predicted, with 112 target genes obtained from the 33 DEMs in MC vs ST, and 456 target genes from the 27 DEMs in ST vs FB. To understand the functions of these genes targeted by DEMs, GO annotation, and KEGG enrichment was performed. Target genes were classified into cell cycle-related, cyanoamino acid metabolism, and energy metabolism-related pathways (Fig. 6A,B). These results indicated that milRNAs played important roles in the growth process of *O. sinensis*.

There were 38 and 75 DEM-DEG relationship pairs found in MC and FB stage with ST as a control, respectively (Table S5). The network regulation diagram drawn by Cytoscape of some functionally annotated target genes indicated that one DEM could regulate more than one DEG, with both positive and negative correlation. Most milRNAs had more than one possible target gene, while different milRNAs could also regulate the same targets. As miRNAs regulate gene expression mainly by promoting cleavage of the target mRNAs or regulating transcription factors (TFs), we focused on negatively correlated pairs. According to the target regulation map in Fig. 6C,D, key enzyme genes in *β* oxidation gene-G6O67_007081 (3-hydroxyacyl-CoA dehydrogenase, targeted by n_os_milR90) and ecological adapting-related gene gene-G6O67_007081 (tat pathway signal sequence, targeted by n_os_milR16) were upregulated. From the ST to FB stage, gene-G6O67_006617 (ABC transporter) and gene-G6O67_008466 (SET domain protein) were significantly downregulated by n_os_milR34, with a log_2_(fold change) of 5.106 and 3.096, respectively. According to the target gene annotation and regulatory network, n_os_milR16, n_os_milR21, n_os_milR34, and n_os_milR90 represent candidate milRNAs to affect fruiting body development.

### Validation of the DEGs and DEMs by RT-qPCR

**Figure 7:**
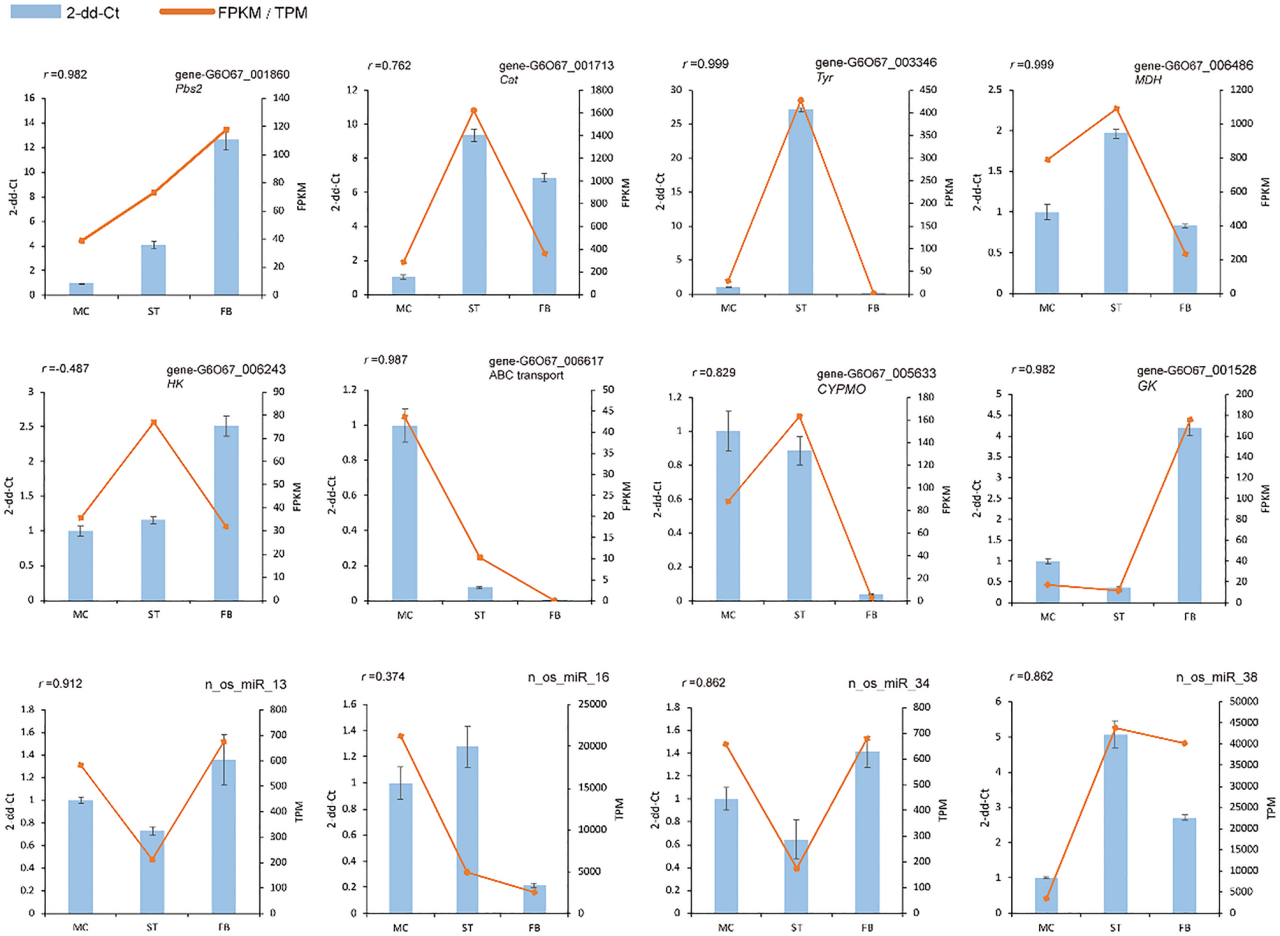
Quantitative RT-PCR (qRT-PCR) validation of DEGs and DEMs at different developmental stages (MC, ST, and FB). The x-axis represents sample names, the left y-axis represents relative expression level (2^-dd-Ct^), and the right y-axis represents RNA-Seq results (FPKM/TPM). ‘*r*’ indicates the Pearson correlation coefficient.

To confirm the reliability of the sequencing data, a total of eight DEGs and four DEMs were randomly selected to validate the RNA-Seq and small RNA expression profiles. As expected, qRT-PCR results showed that most of these mRNAs and miRNAs shared a similar expression with those from the sequencing data. Pearson correlation also showed that most of the relative expression levels were strongly correlated with FPKM/TPM, 83.33% *r*^2^ > 0.8 (Fig. 7), which confirm the reliability of the transcriptome sequencing data described above.

## Discussion

In order to determine the mechanism of induction of fruiting body in *O. sinensis* and analyze the expression of key genes, we performed an integrated mRNA and milRNA profiling of three developmental stages of *O. sinensis* using high-throughput sequencing. Our results provide new insights into the developmental regulation and mechanism of induction of fruiting body.

In the research of the developmental molecular mechanism of the fruiting body among *Neurospora crassa*, *Sordaria macrospora*, *Aspergillus nidulans,* and other model ascomycetes, PKA and MAPK^19–24^ were identified as pivotal signaling pathways. The development of *C. militaris* (FB) and *O. sinensis* (FB) belong to the genus *Cordyceps* is also mainly regulated by these signaling pathways^11,25,26^. Transcriptional profiling indicates that the development of fruiting body was shown to be more dependent on the MAPK signaling pathway than on PKA signaling^27^. Previous research has shown that the MAPK pathway participates in many physiological and developmental processes, including osmotic and oxidative stress, cell and sexual cycle regulation, and virulence^26,28^. MAPKs have also been generally conserved in all species studied thus far, and have very similar organization and functions^20^. Therefore, understanding the regulatory changes of MAPK signaling pathway is of great significance for revealing the differentiation and growth mechanism of the *O. sinensis* fruiting body.

**Figure 8:**
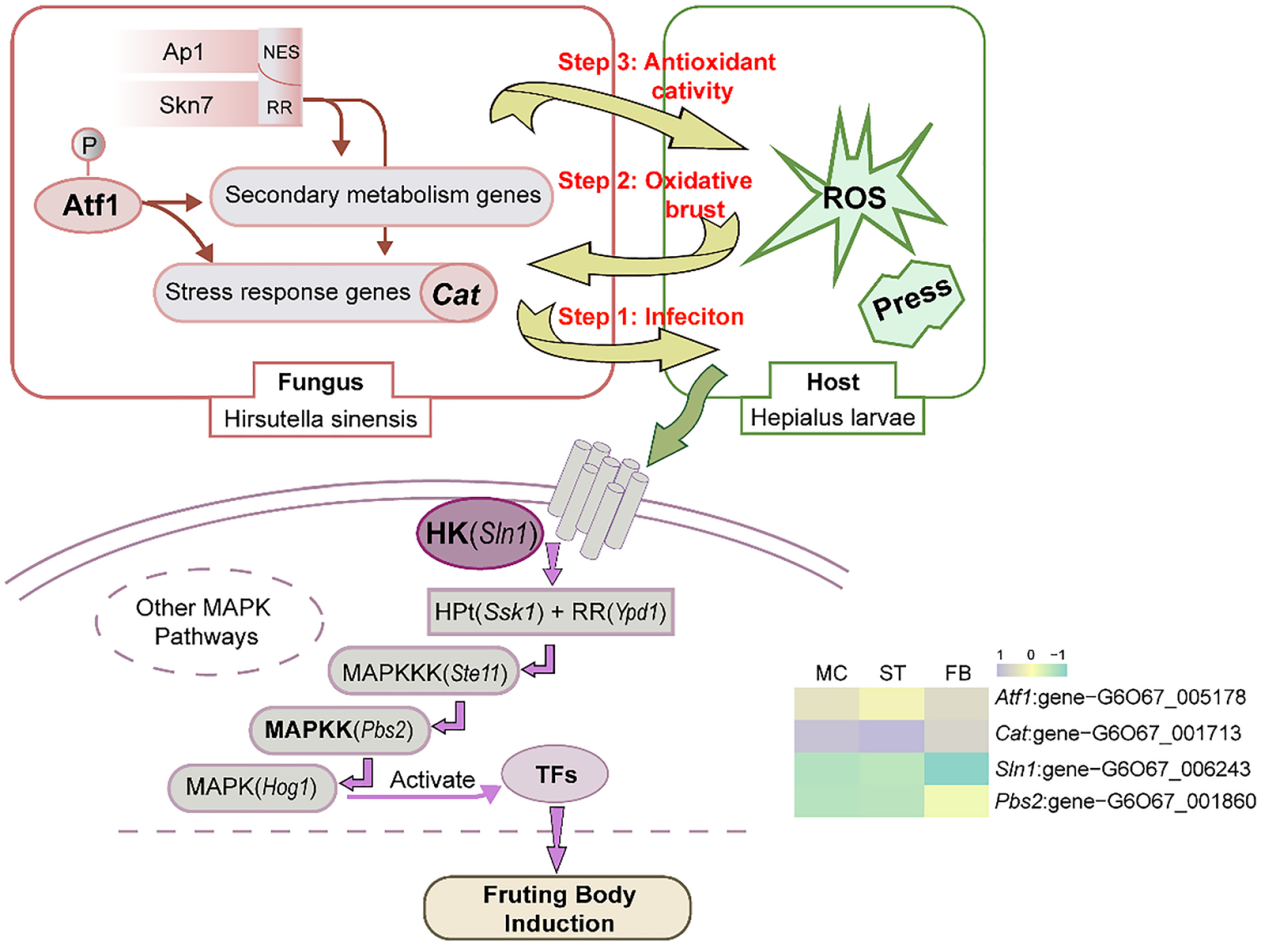
The inferred mechanism of *O. sinensis* fruiting body induction. *atf1*, a transcript factor belongs to the ATF/CREB protein family; *cat1*, catalase; *sln1*, a histidine kinase; *pbs2*, mitogen-activated protein kinase kinase.

The signal transduction processes in which MAP kinases are involved starts with the sensing of environmental stimuli by two-component signal transduction systems (TCS) and receptor tyrosine kinases (RTKs). TCS consists of three components or signal transducers: a histidine kinase (HK, such as *sln1*), a response regulator (such as *ypd1*), and a histidine-containing phospho-transmitter (HPt, such as *ssk1* or *skn7*)^20,23,24,26,29,30^. From the MC to ST stages, the host Hepialus larvae acts as a growth container for *O. sinensis* mycelium, splitting and proliferating continuously in it^31^. The increase in body filling leads to increased pressure in the surrounding cells, which induces autophosphorylation of *sln1* , then subsequently transmitting the stress signal from *sln1* to *ssk1* through *ypd1*, thereby activating MAPK^22,32,33^. On the other hand, oxidative stress can activate different signal transduction pathways, either via the localization of specific regulators to the nucleus upon stress, followed by subsequent activation of detoxification genes expressions, or phosphorylation-driven intervention of the MAPK pathway^20,34,35^. In the MAPK signaling pathway, phosphorylation occurs at every step of signal transduction^36^. The phosphorylation of HK or RTKs directly activates a MAPKKK, which in turn activates a MAPKK via the phosphorylation of serine/threonine residues. This latter protein phosphorylates one or several MAPKs on serine/threonine/tyrosine residues, which finally gives rise to the activation of TFs that induce or repress genes involved in cellular adaptation or response to the sensed stimuli^37,38^. Moreover, DEG analysis also showed that the degree of phosphorylation was highest at the ST stage. Hence, high oxidative stress may activate the MAPK signaling pathway to regulate the formation of *O. sinensis* fruiting body, the inferred induction mechanism is shown in Fig. 8.

Although Li et al. have proposed in the transcriptome study on the sexual development of *O. sinensis*, the high-expression mating-genes indicating fruiting body was initiated in the ST stage. Here, we conducted by collecting the latest two stages of ST, added the important stage of the mycoparasite complex, verified this conclusion from the perspective of MAPK signal pathway. MAPK signaling in *O. sinensis* was most active at the ST stage, but it decreased during the FB period. We speculate that the MAPK signaling pathway is closely involved in the ability of *O. sinensis* to successfully bud and differentiate into fruiting body. Oxidative stress and inflation pressure are the major factors that stimulate MAPK signaling and induce downstream gene expression in fruiting body differentiation, morphogenetic processes, filamentous growth, mating, and osmolyte synthesis.

After fungal infection, the host produces huge amounts of reactive oxygen species (ROS)^39^. Through the mRNA-Seq of *O. sinensis*, we found that a large number of genes related to oxidative activity were highly expressed in the sclerotium stage (Figs. 3, 4, Table S2). The most enriched DEG was the *cat1* which plays a central role in defense against oxidative stress. Catalase can catalyze the breakdown of H_2_O_2_ into O_2_ and H_2_O to protect proteins from oxidation by oxygen^40^. *O. sinensis* has the highest degree of ROS expression and the strongest oxidative stress capacity in the sclerotia stage. Therefore, *O. sinsensis* can eliminate active oxygen through catalase to ensure the survival of fungal cells.

The large amount of reactive oxygen produced by fungi infecting the host can not only induce transcriptional activation of stress response genes but can also activate the biosynthesis of certain secondary metabolites^41,42^. In *Aspergillus*, the antioxidant enzymes were inferred as the first line of defense against excessive ROS formation, while the synthesis of secondary metabolites as the second line of defense against ROS damage^43^. For example, aflatoxins have oxidative stress-inducing properties, which can be inhibited by phenolic antioxidants^41,44^. Through analysis of the chemical components of *O. sinensis*, a large number of antioxidant metabolites were found, such as cordycepic acid, phenols, and vitamin B (riboflavin)^45^, and pathways involved in the biosynthesis of antibiotics were relatively active in the sclerotium stage with a high oxygen environment. Therefore, we speculate that *O. sinensis* can overcomes oxidative breakdown through two ways of antioxidant activity and the production of secondary metabolites as a scavenging for reactive oxygen. Based on the changes in molecular regulation during the development of *O. sinensis*, the overlap between oxidative stress and secondary metabolism may be interpreted as an adaptive mechanism resulting from the molecular dialog between the host and the fungus^39,41^ (Fig. 8).

Most fungal vegetative hyphae do not continuously form fruiting body, but require special nutrient conditions to enable them to acquire a certain stage of “competence” before differentiating the fruiting body^46^. It has been proposed that carbohydrates are stored during vegetative growth to be utilized as a carbon source for sexual development, as well as in preparation for the subsequent fruiting process^47,48^. In our study, energy metabolism, especially carbon metabolism, was most active in the ST stage, prior to the fruiting body formation. In many species, the fruiting body is formed preferentially at much lower nutrient concentrations than those promoting vegetative growth, which may explain why energy metabolism decreases during the FB stage.

With the development of high-throughput sequencing technology, the existence of miRNA or milRNAs in *O. sinensis* have already been shown. Here, 106 novel milRNAs were identified from our sRNA-Seq data, which greatly enrich the miRNA database of this *Cordyceps* fungi^16,18^. miRNAs regulate gene expression mainly by promoting cleavage of the target mRNAs or by regulating TFs; therefore, DEM-DEG target gene relationship pairs can reflect the regulatory function of milRNAs more accurately. For example, the n_os_milR34gene-G6O67_006617 pair, encoding ATP-binding cassette (ABC) transporters, might provide protection against plant defense compounds and fungicides by ATP-driven efflux mechanisms. The defense function of *O. sinensis* was reduced during the FB phase (Fig. 7, Table S6), consistent with the transcriptome analysis (Table S3), implying that one of the factors that induce fruiting body formation might be the reduction of self-defense via the ABC transporter. However, the authenticity of these novel milRNAs requires further verification.

In conclusion, transcriptome and small RNA sequencing analyses of *O. sinensis* were carried out at various development stages. Our results indicated that the critical period for the successful formation of fruiting body was in the sclerotium stage. High oxidative stress and expansion pressure in the sclerotium stage stimulates the MAPK signaling pathway and induces downstream gene expression to promote transcription of genes involved in fruiting body differentiation, morphogenesis, filamentous growth, mating, and the osmotic pressure response. Based on changes in molecular regulation during the development of *O. sinensis*, an overlap between the antioxidant defense system, the catalase enzyme system, and secondary metabolites has been described. Further investigation will be required to verify the function of milRNAs whose expression changed the most during FB stage. Additional functional analysis of DEGs and DEMs would provide critical clues to reveal the molecular mechanism in the development of fruiting body. These target genes or milRNAs may be useful for improving the cultivation of *O. sinensis*.

## Materials and methods

### Fungi materials

Fungi at three developmental stages were collected before and after the induction of fruiting body (Fig. 1). Larvae of the bat moth completely infested by *Hirsutella sinensis* were designated as the mycoparasite complex (MC). Mummified larvae coated with mycelia before stroma development were designated as the sclerotium (ST). Samples of the fruiting bodies were designated as the whole *O. sinensis* with fruiting body (FB). Fresh samples were harvested from the artificial cultivation workshop at Chengdu Eastern Sunshine Co. Ltd, and stored at -80℃ until further processing. The strains of *H. sinensis* were preserved at Sichuan Center of Industrial Culture Collection under the accession number: SICC 5.02.

### RNA extraction, library construction, and sequencing

Total RNA was extracted using the RNeasy Plant Mini Kit (Qiagen, Germany) according to the manufacturer’s protocol. RNA concentration and integrity were evaluated using a Nanodrop2000 (Thermo Fisher Scientific, Wilmington, DE) and Bioanalyzer 2100 system (Agilent Technologies, CA, USA). OD values between 1.8–2.2 and RIN ≥ 7.0 were required, and the concentration of the RNA was not less than 250 ng/μl.

For transcriptome sequencing, 1 μg of total RNA per group was used as input material for RNA sample preparation using a NEBNext Ultra Directional RNA Library Prep Kit for Illumina (NEB, USA). For small RNA sequencing, 5 μg of total RNA was ligated to 5′-RNA and 3′-RNA adaptors according to the NEBNext Multiplex Small RNA Library Prep Set for Illumina protocol (NEB, USA). RNAs were reverse transcribed to cDNAs to obtain a cDNA library, followed by PCR amplification. Two kinds of libraries for sequencing were generated; index codes were added to attribute sequences to each sample, and then samples were sequenced by Biomarker Technology Co., Ltd. (Beijing, China) on an Illumina NovaSeq6000 platform with 125 bp paired-end and 50 bp single-end reads, respectively. Three biological replicates were performed for each sample.

### Analysis of differentially expressed genes (DEGs)

To control the quality of RNA-Seq raw data, the Fast QC Toolkit v0.11.9 (https://www.bioinformatics.babraham.ac.uk/projects/fastqc/) was used to remove adaptor sequences and low-quality reads. The expression level of each transcript was measured as the number of clean reads mapped to its reference sequence. Clean reads from each sample were mapped to the reference genome of *O. sinensis* (NCBI accession number: PRJNA608258) using HISAT2 v2.0.4 (http://daehwankimlab.github.io/hisat2/). StringTie v2.1.2 (https://ccb.jhu.edu/software/stringtie/) was employed to calculate expression levels of genes^49^. Fragments per kilobases of exon per million fragments mapped (FPKM) values were used to normalize the expression level, and differential expression analysis was performed using the DESeq2 v1.30.1 R package (https://bioconductor.org/packages/release/bioc/html/DESeq2.html)50. A False Discovery Rate (FDR) ≤ 0.05 & |log2(fold change, FC)|≥ 1 were set as thresholds for DEG selection.

### Identification of miRNAs and target gene prediction

As there are no miRNAs in miRbase 21.0 (http://www.mirbase.org/) confirmed to be encoded by fungi, approaches to identify animal or plant miRNAs were employed to identify fungal miRNAs or milRNAs^50^. Small RNA raw data in fastq format were first processed through cutadapt and fastp to obtain clean data, excluding reads with an “N” content ≥ 10%, reads without a 3′-adaptor sequences, low-quality reads, and sequences shorter than 18 nt or longer than 30 nt. Bowtie software was used to map the unannotated reads to the reference genome^51^. Mapped reads were aligned with mature miRNA sequences in the miRbase database to identify known miRNAs. miDeep2 (https://www.mdc-berlin.de/content/mirdeep2-documentation) was used to predict new miRNAs from unidentified miRNA reads^52^. Moreover, miRNA target genes were predicted using miRanda and targetscan scripts with default parameters^53^. The expression levels of miRNAs in each sample were normalized using the TPM algorithm. Differentially expressed miRNAs (DEMs) between samples were identified using the DESeq2 R package, with a significance threshold set to FDR ≤ 0.05 and |log2 (fold change)|≥ 1. Cytoscape v3.8.2 (https://cytoscape.org/) software was used to construct a DEMs-DEGs regulatory network^54^. The intersection of “candidate target genes” and s “DEGs” was referred to as “differential target genes”.

### Functional annotation and KEGG pathway enrichment

Gene ontology (GO, http://geneontology.org/) functional annotations and Kyoto Encyclopedia of Genes and Genomes (KEGG, https://www.genome.jp/kegg/) signaling pathway annotations for DEGs and DEMs in *O. sinensis* were performed^55,56^. GO and KEGG annotations were subjected to Fisher’s exact tests (FDR < 0.05) and KOBAS3.0 (http://kobas.cbi.pku.edu.cn/kobas3) with an enrichment *P *value < 0.05, respectively. Moreover, Gene Set Enrichment Analysis (GSEA)^57^ v4.1.0 (http://www.gsea-msigdb.org/gsea/index.jsp) was performed to fully understand the gene expression trends of the gene sets identified in the KEGG or GO term analyses.

### Validation of quantitative real-time PCR (qRT-PCR)

To validate the RNA-Seq and miRNA profiling results, quantitative real-time PCR (qRT-PCR) was performed using a CFX96 Real-time system (Bio-RAD, USA) and carried out with 2X Ultra SYBR Mixture (TransGen, Beijing, China) according to the manufacturer’s instructions. Total RNA samples were the same as the Illumina HiSeq sequencing input samples. Approximately 1 µg RNA from each sample was used to synthesize single-stranded miRNA and cDNA via reverse transcription using the miRcute miRNA first-strand cDNA synthesis kit (TIANGEN, Beijing, China). Ten miRNA-specific and target-gene primers are listed in Table S6. miRNA and target gene expression levels were calculated using the 2^−ΔΔCt^ method and normalized to the levels of 18S ribosomal RNA (rRNA)^58^, each reaction was performed in triplicate^59,60^.

## Supplementary Information

Supplementary Information.

Supplementary Information.

Supplementary Information.

Supplementary Information.

Supplementary Information.

Supplementary Information.

## Data Availability

All data were deposited in the National Center for Biotechnology Information (NCBI) Sequence Read Archive under the accessions GSE160504 (RNA-Seq) and GSE160506 (small RNA sequencing).
